# Spontaneous regression of immunoglobulin G4-related dacryoadenitis and multiple organ involvement: A case report

**DOI:** 10.1097/MD.0000000000032618

**Published:** 2023-01-06

**Authors:** Mayari Ito, Aric Vaidya, Hirohiko Kakizaki, Yasuhiro Takahashi

**Affiliations:** a Department of Oculoplastic, Orbital & Lacrimal Surgery, Aichi Medical University Hospital, Nagakute, Aichi, Japan; b Department of Oculoplastic, Orbital & Lacrimal Surgery, Kirtipur Eye Hospital, Kathmandu, Nepal.

**Keywords:** dacryoadenitis, extra-ophthalmic lesion, Immunoglobulin G4, spontaneous regression, steroids

## Abstract

Immunoglobulin G4 (IgG4)-related dacryoadenitis is rarely resolved spontaneously without steroids. Here, we report a case of IgG4-related dacryoadenitis and extra-ophthalmic lesions with spontaneous regression.

**Methods:**

This is a clinical case report. A 56-years-old man had a 1-year and 7-month-old histories of neck and eyelid swelling, respectively. On the first examination, the lacrimal and submandibular glands were palpable bilaterally. Computed tomographic images showed enlargement of the lacrimal gland on both sides, right pulmonary hilar lymph node, and pancreas, and thickening of the abdominal aortic wall. Blood tests demonstrated elevated serum IgG4 level and positive hepatitis B surface antibody. Pathological examination of the biopsied lacrimal gland specimens revealed marked IgG4-positive plasma cell infiltration.

**Results:**

The patient was monitored carefully without steroid administration. Serum IgG4 level had gradually decreased during follow-up period and reached the normal range 3 years after the biopsy. At 4-year follow-up, the lacrimal and submandibular glands were not palpable on either side. Computed tomographic images demonstrated no enlargement of the lacrimal gland, submandibular gland, or lymph nodes, and improvement of the enlarged pancreas and thickened abdominal aortic wall.

**Conclusion:**

Our case indicates that careful observation can be an option in selected cases with risks of steroid treatment or silent clinical course.

## 1. Introduction

Immunoglobulin G4-related ophthalmic disease (IgG4-ROD) is a relatively common entity of Immunoglobulin G4 (IgG4)-related disease (IgG4-RD).^[1]^ Although the involved tissues include the lacrimal gland, orbital fat, extraocular muscles, trigeminal nerve, eyelid, sclera, lacrimal sac, and optic nerve,^[1–4]^ the lacrimal gland involvement (dacryoadenitis) is most commonly encountered.^[1]^ Concomitant extra-ophthalmic IgG4-RD is sometimes seen.^[1]^ Steroid administration is the first-line treatment for IgG4-ROD, but a considerable number of patients with IgG4-ROD experience relapse after tapering of steroids.^[5,6]^

In contrast, although rare, IgG4-related dacryoadenitis is spontaneously resolved without steroids.^[7,8]^ There have been only a few reported cases of spontaneous regression of IgG4-related dacryoadenitis.^[7,8]^ Here, we report an additional case of IgG4-related dacryoadenitis and multiple organ involvement with spontaneous resolution.

## 2. Case report

This study was conducted in accordance with the tenets of the Declaration of Helsinki and its later amendments. Written informed consent for publication of identifiable face photos was obtained from the patient.

A 56-years-old man presented with a 1-year history of bilateral neck swelling. The patient underwent biopsy of the submandibular gland lesions at an ENT clinic, and malignancy was ruled out. Bilateral eyelid swelling was noticed by his friends 3 months later. The patient denied any history of ocular or systemic diseases and the family history was not significant.

**Figure 1.**
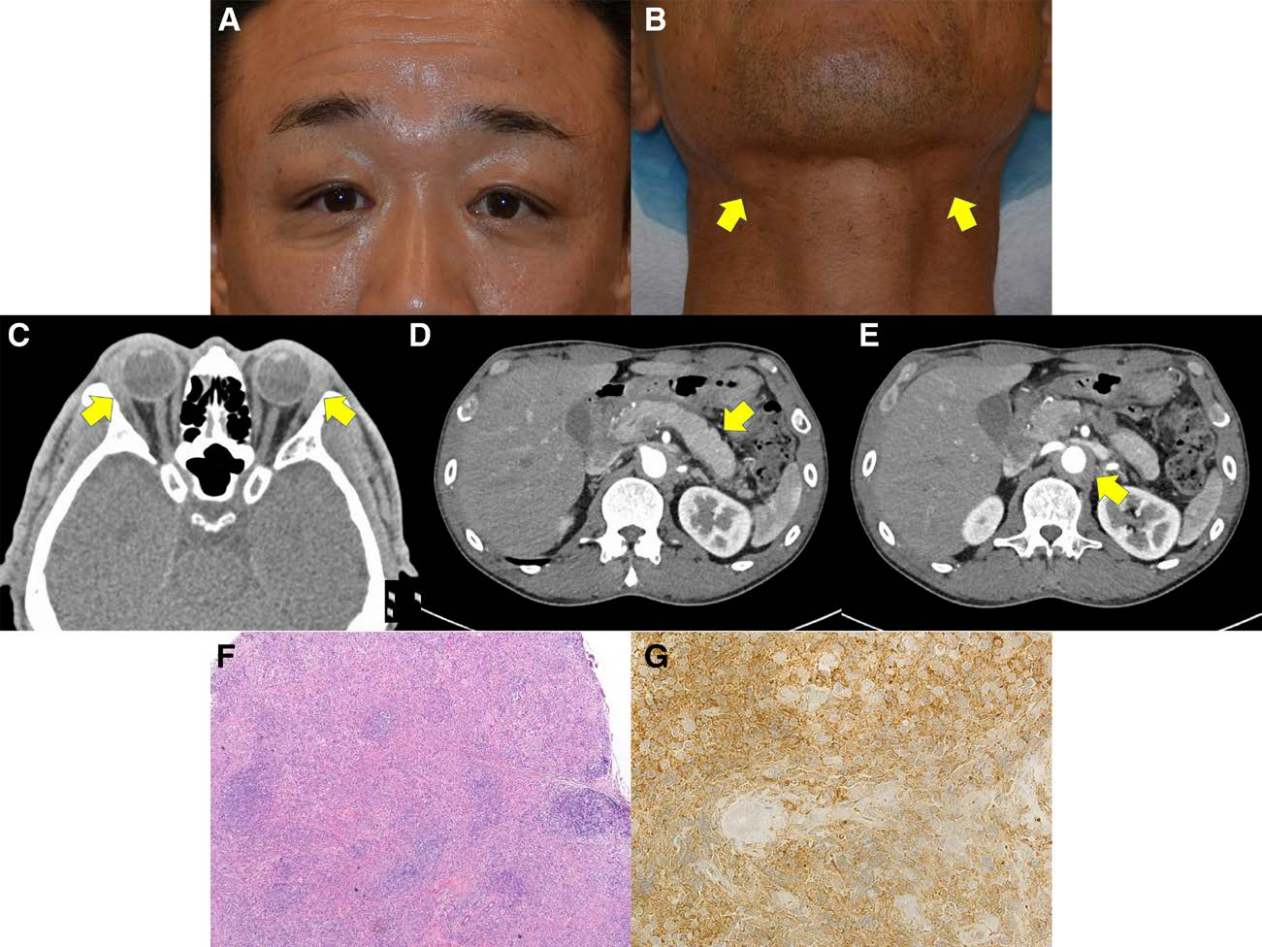
Initial clinical findings of the patient. (A, B). Photos of the patient taken before biopsy. The upper eyelids (A) and submandibular regions (B) (arrows) are swollen on both sides. (C–E). Enhanced axial computed tomographic (CT) images through the orbit and abdomen reveals bilateral lacrimal gland enlargement (C) (arrows), diffuse enlargement of the pancreas with partial effacement of the lobular contour (D) (arrow), and thickening of abdominal aortic wall (E) (arrow). (F, G). Pathological findings. A hematoxylin-and-eosin-stained section showing marked inflammatory cell infiltration (F) (magnification, × 40). Immunohistochemistry for Immunoglobulin G4 (IgG4) revealing IgG4-positive cells infiltration (G) (magnification, × 400). CT = computed tomographic.

On the first examination, his best-corrected visual acuity was 1.0 in both eyes, and intraocular pressure was 12 mm Hg. The lacrimal and submandibular glands were palpable on both sides (Fig. 1A and B). Orbital computed tomographic (CT) images showed bilateral lacrimal gland enlargement (Fig. 1C). Systemic CT images revealed enlargement of the right pulmonary hilar lymph nodes, diffuse enlargement of the pancreas with partial effacement of the lobular contour (Fig. 1D), and thickening of the abdominal aortic wall (Fig. 1E). Blood tests revealed elevated serum IgG level (2048 mg/dL), IgG4 level (1080 mg/dL), soluble interleukin-2 receptor (898 U/mL), immunoglobulin E level (805 IU/mL), and antinuclear antibody (1:40). Thyroid-related autoantibodies, anti-Sjögren-syndrome-A and Sjögren-syndrome-B antibodies, myeloperoxidase- and proteinase 3-antineutrophil cytoplasmic antibodies, and angiotensin-converting enzyme were within the normal range. Hepatitis B surface antibody was found to be positive.

Biopsy of the lacrimal gland lesions on both sides was performed. Pathological examination of the harvested specimens revealed marked inflammatory cell infiltration with fibrosis (Fig. 1F). Lymphoid follicles with germinal centers were also found. IgG4 immunostaining revealed IgG4-positive plasma cells (Fig. 1G), with > 50 IgG4-positive plasma cells per high-power field and a ratio of > 40% IgG4/IgG. These findings were compatible with IgG4-ROD.^[9]^

We carefully observed the patient without steroids because hepatitis B surface antibody was positive and eyelid swelling became inconspicuous after the biopsy. The patient was consulted with a pulmonologist, hepato-biliary-pancreatic physician, and cardiovascular surgeon. The patient refused any further examination and treatment at that time as he was symptomless.

**Figure 2.**
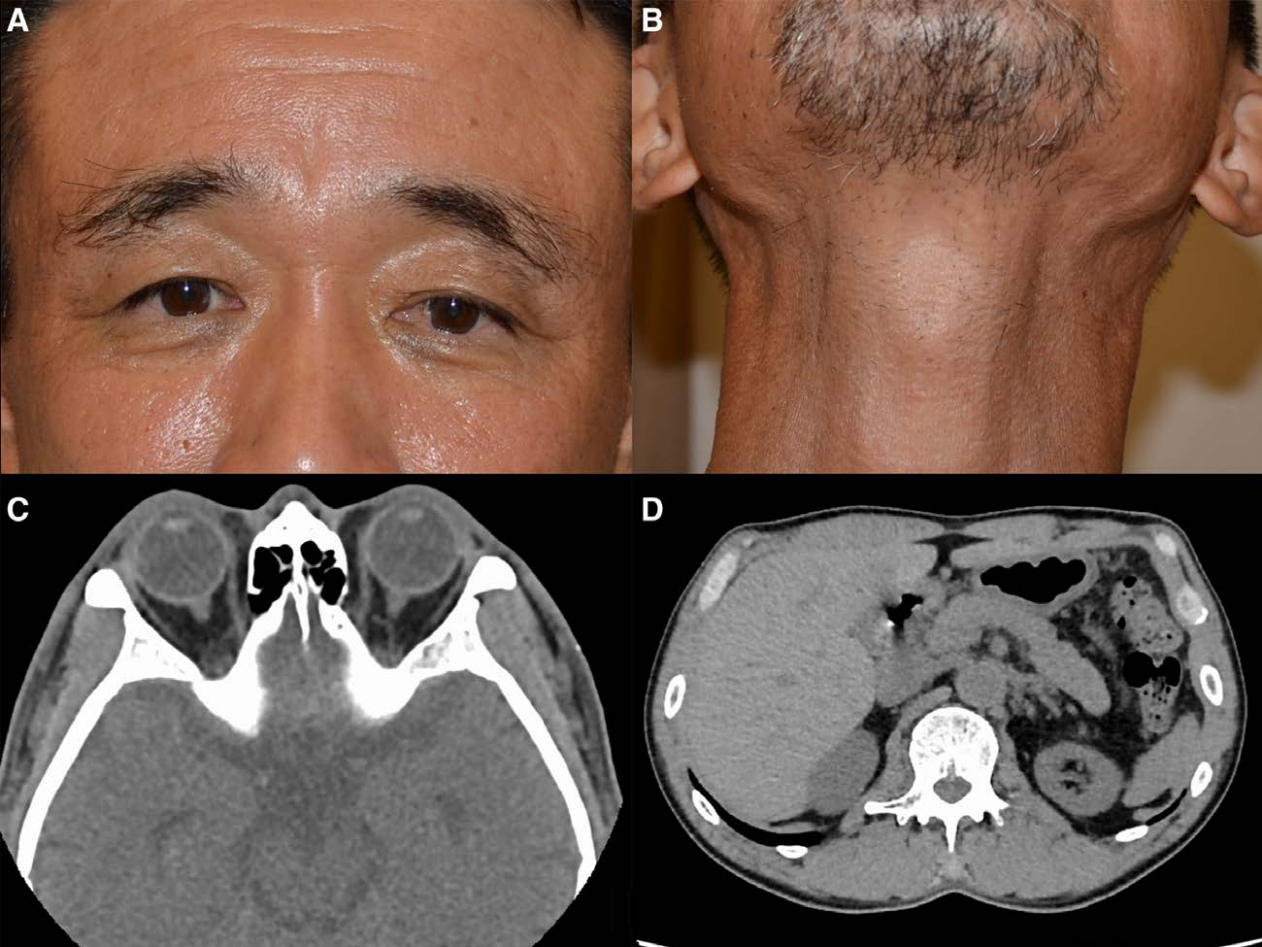
Clinical findings at 4-year follow-up period. (A, B). Photos of the patient. Swelling of the upper eyelids (A) and submandibular regions (B) subside. (C). An axial orbital CT showing no enlargement of the lacrimal gland on either side. (D). An axial abdominal CT showing improvement of the enlarged pancreas and thickened abdominal aortic wall. CT = computed tomographic.

At 4-month follow-up, the lacrimal and submandibular glands were not palpable on either side. CT images taken at 2-year follow-up period revealed improvement of lesions of the lymph nodes, pancreas, and aortic wall, and endoscopic ultrasound-guided fine needle aspiration of the pancreatic lesion showed no inflammatory cell infiltration. Serum IgG4 level had gradually decreased during the follow-up period and reached the normal range (110 mg/dL) 3 years after the biopsy. At 4-year follow-up, the lacrimal and submandibular glands remained non-palpable on both sides (Fig. 2A and B), and CT images demonstrated no enlargement of the lacrimal gland (Fig. 2C), submandibular gland and lymph node, and improvement of the enlarged pancreas and thickened abdominal aortic wall (Fig. 2D).

## 3. Discussion

We report a case of IgG4-related dacryoadenitis and other extra-ophthalmic lesions with spontaneous regression. Although IgG4-related submandibular and pancreatic lesions occasionally regressed spontaneously without treatment,^[8,10]^ spontaneous regression of IgG4-related dacryoadenitis is a rare event. A previous report showed a case of sole IgG4-related dacryoadenitis with spontaneous regression.^[7]^ Another report presented that 1 of 27 untreated patients with IgG4-RD obtained spontaneous improvement of the lacrimal gland lesion; however, the detailed clinical findings were not presented.^[8]^

The mechanisms of spontaneous regression remain unclear. A previous report postulated that decreased absolute amount of IgG4-positive plasmacytes after biopsy induces an abnormal immune response.^[11]^ This may decrease serum IgG4 level and further reduce the size of the biopsied lacrimal gland.^[7]^ Another proposed mechanism is alteration of cellular immunity after biopsy, which may lead to regression of other lesions that are not biopsied.^[7]^ Since serum IgG4 level gradually decreased and all of the lesions regressed after biopsy of the lacrimal gland lesions, those postulated mechanisms may be applicable to our case.

Previous studies demonstrated that sex, serum levels of C3, C4, and CH50, new-onset diabetes mellitus, and extensive multi-organ involvement were factors influencing the clinical course of patients with IgG4-RD without treatment.^[8,10,12]^ Additionally, high serum levels of IgG and IgG4, and Mikulicz disease also cause poor clinical course of IgG4-ROD during steroid treatment.^[5,6,13]^ We did not examine serum levels of C3, C4, and CH50 in our case. However, our case had spontaneous regression of IgG4-related lesions, even though male sex, extensive multi-organ involvement, high serum levels of IgG and IgG4, and bilateral lacrimal and submandibular glands enlargement (Mikulicz disease) were considered as negative influential factors in the clinical course of IgG4-ROD and IgG4-RD. This case indicates that spontaneous regression of IgG4-ROD and -RD can be expected even in cases with risk factors for poor clinical course of these entities.

Spontaneous regression of multiple lesions in this case suggests that the wait-and-watch policy can be an option in selected cases with risks of steroid treatment or a silent clinical course. However, careful monitoring is necessary as a prolonged clinical course may cause dysfunction of the involved organs.^[8]^ Other treatment options, including other medications, radiotherapy, and debulking^[5]^ should be considered when lesions deteriorate during observation.

In conclusion, we report a rare case of IgG4-related dacryoadenitis and extra-ophthalmic lesions that spontaneously regressed without steroids. Although wait-and-watch policy can be an option, patients should be carefully monitored for early therapeutic intervention when the lesions deteriorate.

## Author contributions

**Conceptualization:** Yasuhiro Takahashi.

**Formal analysis:** Yasuhiro Takahashi.

**Investigation:** Yasuhiro Takahashi.

**Methodology:** Yasuhiro Takahashi.

**Project administration:** Yasuhiro Takahashi.

**Supervision:** Yasuhiro Takahashi.

**Validation:** Yasuhiro Takahashi.

**Writing – original draft:** Mayari Ito, Yasuhiro Takahashi.

**Writing – review & editing:** Aric Vaidya, Hirohiko Kakizaki.
